# The Abscopal Effect of Radiation Therapy: What Is It and How Can We Use It in Breast Cancer?

**DOI:** 10.1007/s12609-017-0234-y

**Published:** 2017-03-02

**Authors:** Zishuo I. Hu, Heather L. McArthur, Alice Y. Ho

**Affiliations:** 1 Icahn School of Medicine, Mount Sinai Health System, New York, NY USA; 2 Department of Medicine, Cedars-Sinai Medical Center, Breast Oncology, Los Angeles, CA USA; 3 Department of Radiation Oncology, Cedars-Sinai Medical Center, Los Angeles, CA USA

**Keywords:** Breast cancer, Radiotherapy, Abscopal effect, Immunotherapy

## Abstract

The abscopal effect refers to the ability of localized radiation to trigger systemic antitumor effects. Over the past 50 years, reports on the abscopal effect arising from conventional radiation have been relatively rare. However, with the continued development and use of immunotherapy strategies incorporating radiotherapy with targeted immunomodulators and immune checkpoint blockade, the abscopal effect is becoming increasingly relevant in less immunogenic tumors such as breast cancer. Here, we review the mechanism of the abscopal effect, the current preclinical and clinical data, and the application of the abscopal effect in designing clinical trials of immunotherapy combined with radiotherapy in breast cancer.

## Introduction

Dr. RH Mole first coined the term abscopal effect as “an action at a distance from the irradiated volume but within the same organism” in 1953 [1]. In oncology, the abscopal effect refers to the ability of localized radiation to trigger systemic antitumor effects. Over the past 50 years, the abscopal effect arising from conventional radiation has been sparsely reported. A recent review of 23 clinical cases of the abscopal effect after radiotherapy (RT) alone noted that the majority of reported cases occurred in immunogenic tumors such as renal cell carcinoma (RCC), melanoma, and hepatocellular carcinoma (HCC) [2]. However, with the continued development and use of immunotherapy strategies incorporating combinations of targeted immunomodulators and immune checkpoint blockade with radiotherapy, the abscopal effect is becoming increasingly relevant in less immunogenic tumors such as breast cancer. Here, we will review the mechanism of the abscopal effect, the existing preclinical and clinical data, and the use of the abscopal effect in designing clinical trials of immunotherapy combined with RT in breast cancer.

## Mechanism of the Abscopal Effect

**Fig. 1:**
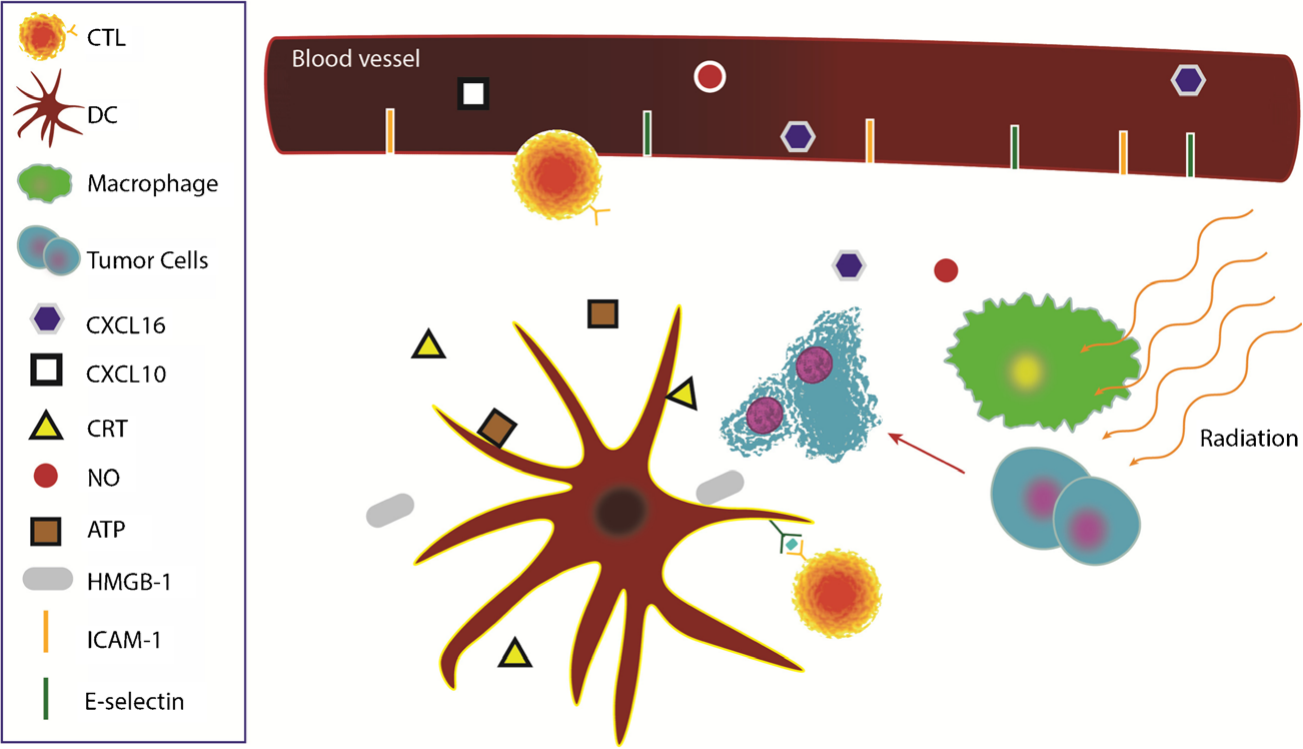
RT causes immunogenic cell death, leading to the release of HMGB-1 and ATP and the translocation of CRT to the cell surface. DCs bind to these molecules to further enhance antigen cross-presentation and CTL priming. RT also promotes release of chemokines CXCL10 and CXCL16 which attract T cells to the tumor. Macrophages also release NO, stabilizing the local tumor vasculature. *CRT* calreticulin, *DC* dendritic cells, *CTL* cytotoxic T lymphocyte, *NO* nitric oxide, *HMGB-1* high-mobility group box 1 protein, *ATP* adenosine triphosphate

The role of RT has historically focused on controlling and eradicating local disease by maximizing direct tumor cell damage and minimizing healthy tissue damage [3]. RT was considered immunosuppressive secondary to reduced blood counts, which were attributable to older techniques that included large amounts of bone marrow and/or circulating blood volume within the radiation portals [4]. The role of total body irradiation prior to stem cell transplantation is an example of this concept in which lymphoablation and myeloablation are induced, secondary to the inherent radiosensitivity of hematopoietic cells [5]. More recently, RT has been shown to promote a number of systemic immune modulatory effects on the tumor as well. The abscopal effect is believed to arise from local RT’s capacity to elicit these systemic immune effects to control unirradiated tumor burden. RT acts as an immune modulator in the tumor microenvironment through several mechanisms which will be elucidated in this review (Fig. 1).

Localized RT induces cell death and release of immunogenic factors via a process termed “immunogenic cell death” (ICD), which subsequently triggers the release of a number of endogenous damage-associated molecular patterns (DAMPs). These DAMPs, which include calreticulin, high-mobility group box 1 protein (HMGB1), and adenosine triphosphate (ATP), contribute to the priming of the immune system by triggering dendritic cells (DCs), thereby resulting in improved antigen presentation to T cells [6, 7]. Specifically, during ICD, dying cells translocate calreticulin to the cell surface and are processed by DCs, facilitating tumor antigen presentation and cytotoxic T lymphocyte (CTL) stimulation [8]. The release of HMGB1 acts as a pro-inflammatory mediator, stimulating monocyte production of the cytokines TNF, IL-1, IL-6, and IL-8 [9]. HMGB1 also improves tumor antigen presentation by binding to Toll-like receptor 4 (TLR4) on DCs and preventing the accelerated degradation of antigens within DCs [4, 5, 10–12]. Released ATP binds to the purine receptors on DCs, leading to inflammasome activation and IL-1β release [13]. Released DNA from dying cells can also activate the stimulator of interferon gene (STING) pathway in DCs, initiating type I interferon (IFN) production and enhancing DC cross-priming [14].

RT has also been shown to stimulate tumor cell release of chemokines CXCL16 and CXCL10, to increase the expression of adhesion molecules E-selectin and ICAM-1 in endothelial cells and to upregulate major histocompatibility complex (MHC1), Fas, ICAM-1, and NKG2D ligands [15–21]. Lastly, RT when combined with adoptive therapy may render tumors accessible to infiltration and help normalize vasculature in the tumor microenvironment [22]. Low-dose radiation has also been reported to recruit NOS2-expressing macrophages to the tumors, subsequently enhancing T cell infiltration and normalizing tumor vasculature [23].

The infrequency of the abscopal effect in the clinical setting is likely due to the counterbalance of the pro-immunogenic signals generated by RT with the immunosuppressive effects of RT. RT promotes TGF-β levels, recruitment of myeloid-derived suppressor cells (MDSCs), and enrichment of regulatory T cells, which play an immunosuppressive role [24–27]. With broader use of targeted immunotherapy agents such as the checkpoint inhibitors, TLR agonists, and cytokines in combination with RT to stimulate the immune system, however, the abscopal effect is likely to be reported with increasing frequency and in less immunogenic tumors.

## Preclinical Reports of the Abscopal Effect

### RT with Immunostimulatory Molecules

Tumors subvert the immune system through a variety of local and systemic processes, including overexpression of T cell inhibitory signals, underexpression of costimulatory signals, promotion of immune suppression of the microenvironment, and lowering antigen presentation. A number of efforts in combining RT with immunotherapy have focused on the strategy of enhancing immunostimulatory signals and blocking inhibitory signals.

A large body of preclinical data has been published on the use of RT with immunostimulatory molecules such as interleukin-2 (IL-2), FMS-like tyrosine kinase 3 ligand (Flt3-L), and Toll-like receptor (TLR) ligands to generate the abscopal effect in mouse models of renal cell carcinoma, breast cancer, and colon cancer.

One of the first preclinical studies on the abscopal effect combined RT with IL-2, a cytokine that plays a central role in lymphocyte activation and proliferation [28]. A mouse model of RCC with pulmonary metastases was given either RT alone, RT with IL-2, or no treatment at all. The group found that localized RT combined with IL-2 eliminated more pulmonary metastases compared to localized RT alone. Yasuda et al. also investigated the use of IL-2 in combination with radiation to treat a mouse model of colon adenocarcinoma with metastases to the liver [29]. Mice were treated locally with either RT, IL-2 alone, RT with IL-2, or no treatment. The mice treated with combined RT and IL-2 showed the largest decrease in the flank tumor size and no metastatic lesions in the liver after 35 days.

Another immunostimulatory molecule that has been used in combination with RT is Flt3-L, a growth factor for dendritic cells. Chakravarty et al. implanted metastatic lung carcinoma cells into the footpads of mice [30]. After 3 weeks, inoculated mice had developed palpable tumors in their feet and micrometastatic foci in the lungs. The foot tumors were then irradiated with 60 Gy with or without intraperitoneally administered Flt3-L. Following irradiation of the mice’s footpads, the number of pulmonary metastases was reduced, compared to treatment with RT or Flt3-L alone. In another study, Demaria et al. implanted mammary cancer cells bilaterally into the flanks of mice [31]. The flanks were then irradiated unilaterally, and Flt3-L was administered. Local RT alone inhibited tumor growth on the irradiated side but not on the unexposed side. When both RT and Flt3-L were given, however, both the irradiated and nonirradiated sides showed delayed growth.

TLR ligands have also been investigated in combination with RT. Physiologically, TLRs recognize DAMPs and pathogen-associated molecular patterns (PAMPs) and play a key role in host defense by enhancing antigen presentation, promoting cytokine production and upregulating costimulatory molecules on DCs [32]. Dewan et al. used the TLR7 agonist imiquimod in combination with RT in a mouse model of cutaneous breast cancer [33]. The group injected breast cancer cells in primary and secondary sites in mice. When imiquimod was topically applied to the primary site alone, tumor growth was inhibited in both primary and secondary tumor sites. When the primary and secondary sites were treated with imiquimod and the primary site was irradiated, this inhibitory effect was further enhanced, resulting in decreased tumor volumes in both the primary and secondary sites.

### RT with Checkpoint Inhibitors

Physiologically, the immune checkpoints serve key roles in moderating the inflammatory response in the setting of infection and self-tolerance. In the tumor microenvironment, these immune checkpoints are often dysregulated and subvert the host’s immune response to the tumor. The two most studied immune checkpoint receptors are cytotoxic T lymphocyte-associated protein 4 (CTLA-4) and programmed cell death-1 (PD-1). CTLA-4 regulates the early stages of T cell activation by limiting activating signals from the T cell co-stimulatory receptor CD28 by competitively binding to shared ligands CD80 and CD86. PD-1 helps limit the inflammatory response of effector T cells in the peripheral tissue through binding of its ligands PD-L1 and PD-L2.

One of the first preclinical investigations combining RT and immune checkpoint blockade used a poorly immunogenic metastatic mammary mouse carcinoma cell line [34]. Anti-CTLA-4 antibodies, RT, the combination of CTLA-4 antibodies and RT, or no treatment were administered to mice injected with 4T1 cancer cells in their flank. Although CTLA-4 blockade alone did not alter tumor growth or survival, the combination of RT and checkpoint blockade delayed growth of the primary irradiated tumor, increased survival, and inhibited lung metastases formation. CTLA-4 blockade with one fraction of 12 Gy gave a statistically significant increase in survival times but not statistically significant primary tumor control compared to RT alone. Two fractions of 12 Gy given at 48-h intervals, however, resulted in complete tumor regression and longer survival in the majority of treated mice. A subsequent study by Dewan et al. investigated the abscopal effect further by using the TSA breast cancer and the MCA38 colon cancer mouse models. The group implanted either TSA or MCA38 cells into the flanks of mice bilaterally [35]. They then irradiated one side of the mice and administered anti-CTLA-4 antibodies. Growth was delayed not only on the irradiated side but also on the nonirradiated side. Treatment with anti-CTLA4 antibodies alone had no effect on the implanted tumors.

Zeng et al. tested anti-PD-1 antibodies with RT in a mouse glioblastoma multiforme model [36]. They implanted glioma cells intracranially into mice and treated the mice with either sham treatment, anti-PD-1 antibody, RT, or RT with anti-PD-1 antibody. Median survival was highest in the RT with the anti-PD-1 antibody group. Similarly, enhanced tumor control and intratumoral T cell infiltration have also been reported in melanoma and breast cancer mouse models treated with a combination of RT and anti-PD-1 antibody [37]. Another group also explored the triplet combination of RT, CTLA-4 inhibition, and PD-L1 in a melanoma mouse model [38•]. They found that while tumors responded to RT and anti-CTLA4, resistance remained common due to upregulation of PD-L1 on melanoma cells. When PD-L1 was added to the resistant melanoma cells, tumor volume decreased further.

## Clinical Reports of the Abscopal Effect

Clinical evidence of the abscopal effect reported by Postow et al. in a metastatic melanoma patient treated with ipilimumab and radiotherapy [39]. While on ipilimumab as maintenance therapy, the patient received 28.5 Gy in three fractions as palliative RT for right-sided back pain from a paraspinal mass. Four months after RT, the paraspinal mass had regressed along with nonirradiated lesions in the right hilar lymph node and spleen. Ten months after RT, repeat CT scan showed stable, minimal disease. Another case report described a metastatic melanoma patient treated with 54 Gy in three fractions in addition to ipilimumab who achieved a complete response of all his metastases, including unirradiated liver and axillary lesions [40]. A phase I/II clinical study of 34 patients with metastatic castration-resistant prostate cancer treated with ipilimumab and 8 Gy fractions on up to three bone metastases reported a complete response in one patient, stable disease in six patients, and prostate-specific antigen declines of ≥50% [41]. Golden et al. recently completed a study of the abscopal effect after administering GM-CSF along with 3.5 Gy × 10 to patients with metastatic solid tumors [42•]. The group reported abscopal responses in 11 of the 41 enrolled patients. Of note, 5 of 14 breast cancer patients had an abscopal response.

## Radiation Dose, Fraction, and Timing

To date, there is no established consensus on the optimal dose, fraction, and timing of RT to combine with immunomodulation to generate an abscopal response based on preclinical studies. Whereas some studies have supported the efficacy of a single dose of radiation ranging from 0.5 to 25 Gy in inhibiting tumor growth, others have demonstrated that standard doses of 2 Gy or smaller hypofractionated doses of 6 or 8 Gy are more effective than a single large dose [20, 23, 43–45].

Lee et al. reported that when mouse melanoma tumors were treated with 5 Gy × 4 over 2 weeks, the tumors initially responded to RT but subsequently relapsed [46]. In contrast, when Dewan et al. used radiation regimens of 20 Gy × 1, 8 Gy × 3, and 6 Gy × 5 in combination with anti-CTLA-4 antibody, they found that the fractionated doses were more effective in inhibiting tumor growth outside of the field of radiation compared to the single-dose regimen [35]. A single institution review of 47 metastatic melanoma patients treated with ipilimumab and radiation found a significant association with abscopal responses with multiple fraction regimens, specifically with radiation fraction sizes of ≤3 Gy [47].

Investigations are ongoing with regard to the optimal sequencing of checkpoint blockade relative to radiotherapy administration. One study reported no association between abscopal responses in either duration from the first dose of ipilimumab to initial radiation treatment or timing of ipilimumab relative to RT [47].

The size of the irradiated target volume may also play in a role in determining the degree of abscopal response. Larger tumors have been hypothesized to release a larger number and variety of neoantigens upon irradiation [48]. However, larger tumors may also shelter deeper hypoxic areas that are immunosuppressive and radioresistant.

## Clinical Trials of Immune Therapy and RT in Breast Cancer

Based on the promising results of preclinical trials demonstrating the enhancement of the abscopal effect with combinations of immunotherapy and RT, several clinical trials testing the combination in breast cancer patients are actively ongoing (summarized in Table 1). The Memorial Sloan Kettering Cancer Center is recruiting patients with metastatic triple-negative breast cancer (TNBC) patients for a treatment regimen combining pembrolizumab with five fractions of 6 Gy [49]. Patients must have at least two tumors measurable by Response Evaluation Criteria in Solid Tumors (RECIST) criteria, one of which must require palliative radiation. Patients with active brain metastases are excluded. The majority of the 10 patients treated to date in the trial have been irradiated at visceral organ sites. The University of Pennsylvania is comparing two radiation schedules of either three fractions of 8 Gy radiation or one fraction of 17 Gy, in combination with tremelimumab and durvalumab, for metastatic breast cancer patients [50]. In a phase 2 trial, the Netherlands Cancer Institute is comparing nivolumab in combination with either one fraction of 20 Gy, cyclophosphamide, low-dose doxorubicin, or cisplatin in TNBC patients [51]. The Peter MacCallum Cancer Centre in Australia is also running a phase 1 study examining the effects of pembrolizumab with one fraction of 20 Gy in oligometastatic breast cancer [52]. A phase 2 study combining an anti-TGF-β blocker with three fractions of 7.5 Gy in metastatic breast cancer of all subtypes is accruing at Weill Cornell and University of California Los Angeles [53]. The Portland Providence Medical Center is also exploring the use of single-dose stereotactic body radiation therapy (SBRT) prior to administration of an OX40 inhibitor in breast cancer patients with metastases to the liver and lungs [54]. Finally, a study of TLR-7 agonist and cyclophosphamide combined with RT (6 Gy × 5 fractions) for breast cancer patients with skin metastases is ongoing at New York University Medical Center [12].

**Table 1:** Clinical trials using CTLA4/PD1/PDL1 inhibitors and RT for breast cancer

Agent	Conditions	Sponsor	Status	Clinicaltrials.gov ID
Radiotherapy with CTLA4 inhibitors				
Tremelimumab with brain irradiation	Breast cancer with brain metastases	Memorial Sloan Kettering Cancer Center	Phase II, recruiting	NCT02563925 [55]
Radiotherapy with PD1/PDL1 inhibitors				
Pembrolizumab and 6 Gy × 5 within 5–7 days	Metastatic TNBC	Memorial Sloan Kettering Cancer Center	Phase II, recruiting	NCT02730130 [49]
Pembrolizumab and hypofractionated RT	Metastatic breast cancer	Abramson Cancer Center of the University of Pennsylvania	Phase I, recruiting	NCT02303990 [56]
Pembrolizumab and 20 Gy × 1 (SABR)	Oligometastatic breast cancer	Peter MacCallum Cancer Centre, Australia	Phase I, recruiting	NCT02303366 [52]
Durvalumab with Tremelimumab and 8 Gy × 3 fractions vs 17 Gy × 1 fraction^a^	Metastatic breast cancer	Abramson Cancer Center of the University of Pennsylvania	Phase I, recruiting	NCT02639026 [50]
Nivolumab given after either 20 Gy × 1, low-dose doxorubicin, cyclophosphamide, cisplatin, or no induction treatment	TNBC	The Netherlands Cancer Institute	Phase II, recruiting	NCT02499367 [51]
Pembrolizumab and SABR^a^	Breast cancer	University of Chicago	Phase I, recruiting	NCT02608385 [57]
Radiotherapy with miscellaneous immunotherapy				
LY2157299 (TGF-β receptor type 1 kinase inhibitor) and 7.5 Gy × 3	Metastatic breast cancer	Weill Medical College	Phase II, recruiting	NCT02538471 [53]
Imiquimod and/or cyclophosphamide with 6 Gy × 5	Metastatic breast cancer	New York University School of Medicine	Phase I/II, recruiting	NCT01421017 [12]
MEDI6469 (monoclonal antibody to OX40) with RT of 15, 20, or 25 Gy to lung or liver metastases	Metastatic breast cancer to the lung and liver	Providence Portland Medical Center	Phase I/II, recruiting	NCT01862900 [54]

*TNBC* triple-negative breast cancer, *RT* radiotherapy, *SABR* stereotactic ablative radiotherapy

^a^Part of a larger trial

## Future Directions

Exploiting the abscopal effect in breast cancer is intriguing, allowing for both local and systemic control of tumor disease burden. Previously, the abscopal effect with RT alone was a relatively rare event. However, with the development of new immunotherapies that further enhance the immune response, the abscopal response is likely to become more clinically meaningful. Current challenges include optimizing radiation doses to maximize immune stimulation, determining the most favorable radiation sequence, defining the optimal combination of immunostimulatory molecules to use alongside radiation, and further neutralizing the immunosuppressive elements that accompany RT. Further progress in the use of abscopal effect in breast cancer will also require the translation of preclinical data into relevant clinically applicable treatments and the development of evidence-based consensus guidelines. For instance, whereas the majority of preclinical models have used mouse models in which radiation is delivered to tumors implanted into subcutaneous tissues, the aforementioned clinical reports of the abscopal effects have largely stemmed from irradiation of visceral metastases [7, 8]. If the abscopal effect is to be harnessed into an effective treatment, multidisciplinary collaboration with radiation oncology, laboratory research, and medical oncology in the optimal design of clinical trials of immunotherapy and RT will be required.
